# OLS4: a new Ontology Lookup Service for a growing interdisciplinary knowledge ecosystem

**DOI:** 10.1093/bioinformatics/btaf279

**Published:** 2025-05-05

**Authors:** James McLaughlin, Josh Lagrimas, Haider Iqbal, Helen Parkinson, Henriette Harmse

**Affiliations:** Samples, Phenotypes and Ontologies Team (SPOT), EMBL-EBI, Wellcome Genome Campus, Hinxton CB10 1SD, United Kingdom; Samples, Phenotypes and Ontologies Team (SPOT), EMBL-EBI, Wellcome Genome Campus, Hinxton CB10 1SD, United Kingdom; Samples, Phenotypes and Ontologies Team (SPOT), EMBL-EBI, Wellcome Genome Campus, Hinxton CB10 1SD, United Kingdom; Samples, Phenotypes and Ontologies Team (SPOT), EMBL-EBI, Wellcome Genome Campus, Hinxton CB10 1SD, United Kingdom; Samples, Phenotypes and Ontologies Team (SPOT), EMBL-EBI, Wellcome Genome Campus, Hinxton CB10 1SD, United Kingdom

## Abstract

**Summary:**

The Ontology Lookup Service (OLS) is an open source search engine for ontologies which is used extensively in the bioinformatics and chemistry communities to annotate biological and biomedical data with ontology terms. Recently, there has been a significant increase in the size and complexity of ontologies due to new scales of biological knowledge, such as spatial transcriptomics, new ontology development methodologies, and curation on an increased scale. Existing Web-based tools for ontology browsing such as BioPortal and OntoBee do not support the full range of definitions used by today’s ontologies. In order to support the community going forward, we have developed OLS4, implementing the complete OWL2 specification, internationalization support for multiple languages, and a new user interface with UX enhancements such as links out to external databases. OLS4 has replaced OLS3 in production at EMBL-EBI and has a backward compatible API supporting users of OLS3 to transition.

**Availability and implementation:**

The source code of OLS is available at https://github.com/EBISPOT/ols4 and DOI 10.5281/zenodo.14960290 with Apache 2.0 License. A freely available implementation is accessible at https://www.ebi.ac.uk/ols4.

## 1 Introduction

The Ontology Lookup Service (OLS) is a search engine for ontologies, first released in 2006 (Côté *et al.* 2006). It supports users to search for ontology terms during knowledge curation required by the Findable, Accessible, Interoperable, and Reusable principles. Users of OLS include high-throughput phenotyping centers producing and exporting their data, such as members of the International Mouse Phenotyping Consortium (IMPC) (Groza *et al.* 2023); data integration initiatives such as the OpenTargets Platform for drug target identification and prioritization (Ochoa *et al.* 2023); and curators of databases including the Genome-Wide Association Study (GWAS) Catalog (Cerezo *et al.* 2025), Expression Atlas (Moreno *et al.* 2022), European Genome–Phenome Archive (Freeberg *et al.* 2022), Polygenic Score Catalog (Lambert *et al.* 2021), ChEMBL (Gaulton *et al.* 2012), WormBase (Harris *et al.* 2010), EuropePMC (Gou *et al.* 2014), PRIDE (Perez-Riverol *et al.* 2025), Ensembl (Dyer *et al.* 2025), IntAct (Hermjakob *et al.* 2004), CancerModels.org (Perova *et al.* 2024), BioStudies (Sarkans *et al.* 2018), and the BioImage Archive (Hartley *et al.* 2022). Recent applications of OLS include harmonization and standardization of data, for example protocols across phenotyping centers; quality control checks on raw data, e.g. correction of data submission errors and detection of baseline drift due to instrumentation; and tracking the progress of phenotyping efforts by funding bodies.

Successive iterations of OLS have evolved in response to user needs, e.g. as standards for Application Programming Interfaces (APIs) changed from SOAP to REST (Côté *et al.* 2010) and when new OBO and Web Ontology Language (OWL) version 2 (OWL2) (https://www.w3.org/2007/OWL/draft/ED-owl2-new-features-20081202/all.pdf) standards for ontologies were introduced (Jupp *et al.* 2015). Recently, the scale and complexity of biological and chemical knowledge have increased dramatically. New methodologies such as spatial transcriptomics have changed the resolution of data to single cell; e.g. OLS is used in the Human BioMolecular Atlas Program (Börner *et al.* 2025); and high performance computing has become more abundant. In turn, ontologies have grown significantly in scale: in December 2016, OLS indexed 158 ontologies with 4 862 923 classes. In December 2024, OLS indexed 266 ontologies with 8 682 322 classes. New authoring tools such as ROBOT templates (Jackson *et al.* 2019) and Dead Simple OWL Design Patterns (DOSDP) (Osumi-Sutherland *et al.* 2017) have expedited this process by making the development of ontologies more automated enabling new terms to be added in large quantities. The complexity of ontologies has also increased; e.g. internationalization to support the translation of ontologies into different languages to support a diverse and international user base (Gargano *et al.* 2024), and features from the OWL2 specification such as disjointness statements and property chains. So far none of the existing open-source solutions [OLS3, BioPortal (Noy *et al.* 2009), OntoBee (Ong *et al.* 2017), and AgroPortal (Jonquet *et al.* 2018)] are able to comprehensively support these use cases.

## 2 Materials and methods

OLS4 is the new version of the Ontology Lookup Service. The OLS4 data-load and backend are implemented in Java 11 and Spring Boot. OLS4 uses Neo4j as a graph database and Solr for full text search. We use Neo4j rather than an RDF triplestore as Neo4j has strong support for recursive queries, used in the OLS tree view and API to retrieve all ancestors and descendants of an ontology node. The labeled property graph (LPG) structure used by Neo4j also enables provenance and reference information attached to OWL axioms to be represented as properties of graph edges. In future, this LPG representation could enable OLS data to be represented and queried using the emerging KGX standard for knowledge graph interoperability (Caufield *et al.* 2023) and incorporated into wider biomedical knowledge graphs such as BioCypher (Lobentanzer *et al.* 2023).

The architecture has been simplified from OLS3; Neo4j is now used as a standalone server rather than an embedded database, and ontology metadata is also stored in Solr removing the need for a MongoDB instance. The motivation for this simplification was to reduce the complexity of deploying OLS outside of its primary instance at EMBL-EBI, for use cases such as the MONARCH Initiative OLS and the NFDI4Chem Terminology Service (Steinbeck *et al.* 2023). The ETL pipeline has also been simplified by eliminating redundant processing to improve scaling of the dataload. OLS4 dataloads for the complete set of ontologies take on average 6% of the time used by OLS3. These faster dataloads allow users to see updates to ontologies more quickly and reflect changes in knowledge, particularly important when biological knowledge develops rapidly which is an important issue for pandemic preparedness; OLS is currently being used as part of the European Viral Outbreak Response Alliance project.

In OLS4, the Neo4j and Solr schemas are dynamic and depend on the annotation properties used in the OWL entities in the source ontologies. OWL entities are translated from RDF to a lossless JSON representation, which is then stored complete and unmodified in both Neo4j and Solr alongside the extracted queryable properties. Queries to Neo4j and Solr include this JSON representation which is used to generate API responses and the frontend pages. In order to maintain backward compatibility, the API is implemented using view classes which match the previous OLS3 data model, but abstract from the underlying OLS4 data model. The use of abstract views enables multiple API versions to be built over the same underlying data model, and changes to the API without reloading data each time to deliver updates delivering new API use cases more quickly to users.

The OLS4 frontend has been rewritten to communicate with the backend exclusively using HTTP APIs rather than by directly accessing the internal data model, offering an option to build new front ends for an OLS4 backend instance, such as third party interfaces tailored to specific ontologies/use cases. Custom instances of OLS3 such as the NFDI4Chem terminology service, which previously had to run divergent OLS instances with locally modified backend code, will in future be able to use the latest OLS backend code coupled to a customized frontend reducing the overhead of keeping the backend code synchronized.

## 3 Results

OLS4 has many new features including full implementation of the OWL2 specification; annotations on annotations; internationalization support; cross-references between ontology terms; and BioRegistry (Hoyt *et al.* 2022) integration. The OWL2 specification has been implemented comprehensively and tested using a suite of test cases based on both the OWL2 Primer and example test-cases extracted from biological and biomedical ontologies, including the Experimental Factor Ontology (EFO) (Malone *et al.* 2009) and the MONDO Disease Ontology (Vasilevsky *et al.* 2022). OLS4 is therefore able to support ontologies using OWL2 features; e.g. in the MONDO disease ontology where OWL2 disjointness is asserted between extrapulmonary tuberculosis (MONDO:0000368) versus pulmonary tuberculosis (MONDO:0006052); and in the Relation Ontology (RO) where the OWL2 property chain regulates=directly regulates ->directly regulates is defined to describe transitive regulation relations. These OWL2 definitions are now visible in the OLS browser and API. In addition to OWL2 ontologies, OLS4 loads schemas defined using rdfs: Class hierarchies, providing users with a standard API to access both OWL ontologies and commonly used schemas such as Dublin Core (https://www.rfc-editor.org/rfc/rfc2413) and Schema.org (Guha *et al.* 2016), which are in turn used by OWL2 ontologies.

OLS4 improves annotation support by implementing annotations on annotations (sometimes termed reification), making references and provenance associated with ontology axioms visible in the web interface. For example, UBERON (Mungall *et al.* 2012) is a widely used multispecies anatomy ontology. The UBERON term for “lung” contains homology notes derived from *The evolution of organ systems* (Schmidt-Rhaesa 2007), a link which is now visible in the corresponding OLS page for attribution and cross-referencing. Full internationalization of annotations has also been added in OLS4; ontologies are browsable in multiple languages and OLS4 displays a language picker listing all languages present in the ontology. When a language is selected, annotations are displayed in the language selected by the user where possible. This functionality has been demonstrated in the Human Phenotype Ontology (HPO) (Gargano *et al.* 2024) which is now accessible in multiple languages in the main OLS instance, enabling curators to map to consistent phenotype terms across language barriers.

**Figure 1.**
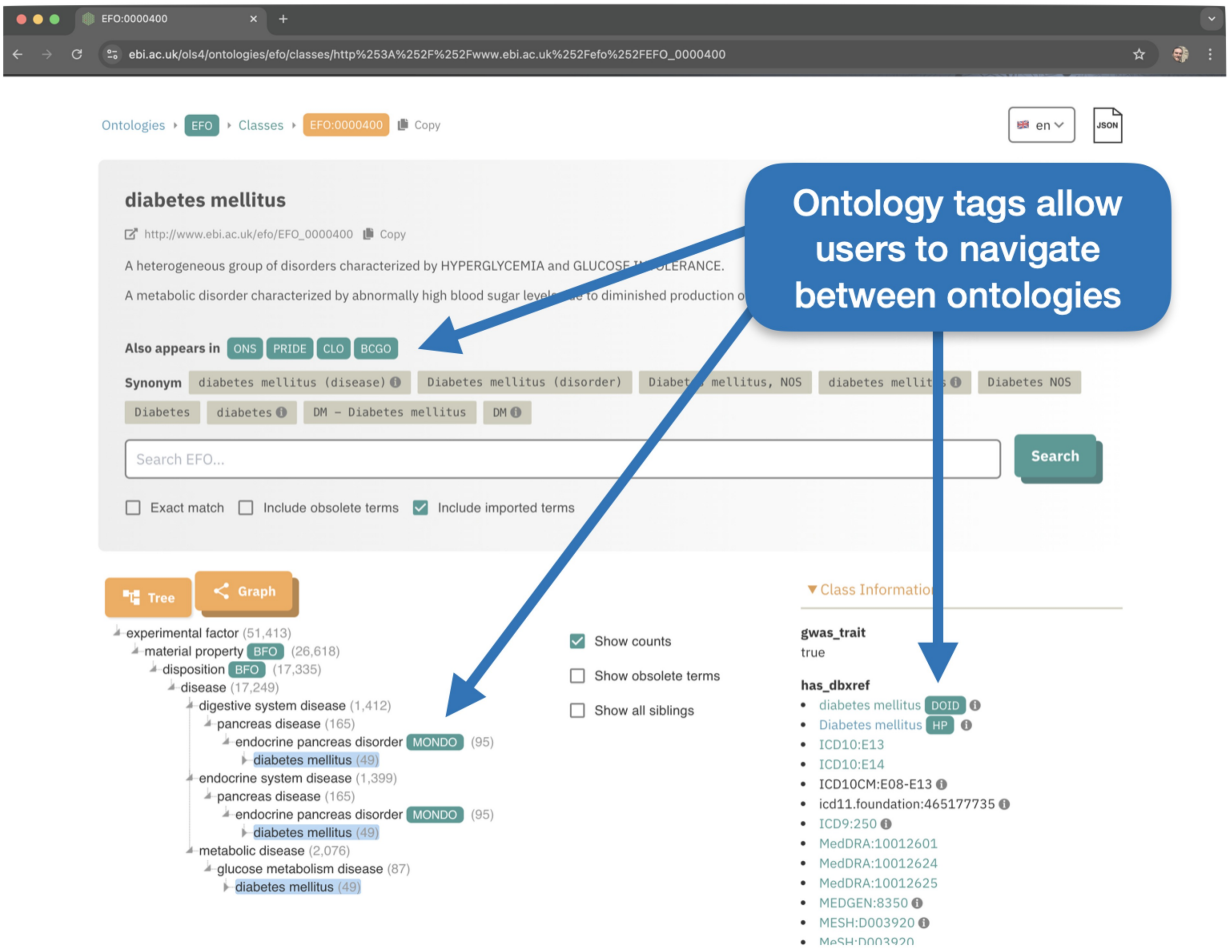
An ontology term from the Experimental Factor Ontology (EFO) viewed in OLS4. References to terms in different ontologies are tagged with links to the corresponding defining ontologies for easy navigation.

Another significant development in OLS4 is the handling of cross-references between ontology terms. The majority of the ontologies indexed by OLS do not exist in isolation, but reuse terms from other ontologies. For example, the EFO imports chemical terms from the Chemical Entities of Biological Interest ontology. In OLS4, such imported terms are labeled in the tree with a tag linking to the defining ontology (Fig. 1). This functionality is critical to support the Unified Phenotype Ontology (uPheno) (Matentzoglu *et al.* 2024), which aggregates terms from multiple phenotype ontologies often with the same name; without the defining ontology tags it would be unclear the difference between, e.g. HPO and MP (Smith *et al.* 2005) terms, in the tree view. In addition to cross-references between ontology terms, OLS4 also automatically creates external links using the Bioregistry (Hoyt *et al.* 2022), enabling users to easily navigate from ontologies to external databases, e.g. from a gene to a genome sequence in GenBank (Sayers *et al.* 2023).

Altogether these features allow OLS4 to deliver a range of new use cases for the ontology community, and to make ontologies more interoperable with other biological and chemical resources. OLS4 is now in production at EMBL-EBI and served approximately 50 million requests from approximately 200 000 unique hosts between November 2023 and February 2024.

## 4 Discussion

Future work will include support for the Simple Standard for Sharing Ontological Mappings (SSSOM) (Matentzoglu *et al.* 2022). Mappings between ontology terms are used, e.g. to map phenotype terms between human phenotypes and model organisms such as mouse and zebrafish. While some mappings are present in ontologies, often represented as hasDbXref properties, SSSOM allows multiple different mapping sets to be defined with associated mapping metadata, which is important as mappings are often subjective and project-dependent. We plan to add support to load and display alternative sets of mappings depending on user preference, e.g. to allow users to choose between different HPO to MP mappings provided by MGI, IMPC, and Pistoia Alliance.

In future, we also plan to implement more sophisticated search capabilities, such as searching for a specific annotation with a specific value and searching in a specific branch of an ontology. For example, EFO terms used to annotate studies in the GWAS Catalog are annotated with a property gwas trait=true. Searching for terms with this annotation would allow the impact of deprecating or moving a term on annotated datasets to be assessed. Limiting a search to a specific branch of an ontology would allow users interested in, e.g. cardiology to limit their searches to terms underneath “heart disease” in MONDO or “heart” in UBERON.

OLS serves curators, annotators, data resource producers, and ontology developers. It has been designed to meet the needs of these user groups as well as to scale for larger and more complex ontologies. For curators and annotators, OLS4 provides a richer view of terms including complete OWL2 axiomatization to help users select appropriate terms and navigate between terms, a unique feature of OLS4 among open source ontology browsers. OLS4 also adds multiple language support which enables bio-curators to search for terms using labels in their native languages, a feature so far only supported by AgroPortal but useful for human disease communities who also need common names. For data resource producers, the faster dataload will enable resources to load and link to the latest versions of ontology terms in days without waiting several weeks for OLS to update. For ontology developers, the new display features of OLS will enable new use cases to be delivered in ontologies with visibility to users, as has been demonstrated for HPO and MP internationalized editions. OLS4 will also help to prevent the proliferation of terms across multiple ontologies re-defining the same concepts in different contexts, by adding ontology tags to make the presentation of the relationship between ontologies more prominent and easier to navigate.
